# The Relationship Between Cervicovaginal Infection, Human Papillomavirus Infection and Cervical Intraepithelial Neoplasia in Romanian Women

**DOI:** 10.3390/diseases13010018

**Published:** 2025-01-16

**Authors:** Anca Daniela Brăila, Cristian-Viorel Poalelungi, Cristina-Crenguţa Albu, Constantin Marian Damian, Laurențiu Mihai Dȋră, Andreea-Mariana Bănățeanu, Claudia Florina Bogdan-Andreescu

**Affiliations:** 1 Department of Obstetrics and Gynecology, University of Medicine and Pharmacy of Craiova, 200349 Craiova, Romania; anca.braila@umfcv.ro (A.D.B.); constantin.damian@umfcv.ro (C.M.D.); laurentiu.dira@umfcv.ro (L.M.D.); 2 Department of Obstetrics and Gynecology, “Carol Davila” University of Medicine and Pharmacy, 020021 Bucharest, Romania; cristian.poalelungi@umfcd.ro; 3 Department of Genetics, “Carol Davila” University of Medicine and Pharmacy, 020021 Bucharest, Romania; 4 Department of Speciality Disciplines, “Titu Maiorescu” University, 031593 Bucharest, Romania; claudia.andreescu@prof.utm.ro

**Keywords:** cervical intraepithelial neoplasia, human papillomavirus, cervicovaginal infection

## Abstract

Cervical intraepithelial neoplasia (CIN) is a premalignant cervical condition closely linked to persistent high-risk HPV infection, a major risk factor for cervical cancer. This study aims to investigate the relationship between cervicovaginal infections, HPV infection, and CIN development in 94 Romanian women with cervical lesions. Comprehensive assessments included HPV genotyping, cytology, colposcopy, and histopathology. In 53.20% of cases, vaginal infections were identified, with *Candida albicans* most frequently associated with HPV. Histopathology revealed 48.94% low-grade CIN, 42.55% high-grade CIN, and 8.51% invasive carcinoma. There was a strong correlation between high-risk HPV types (especially HPV 16 and 18), colposcopic findings, histopathology, and age. This study emphasizes the mutual effect of cervicovaginal infections and HPV infection in increasing the risk of developing CIN and cervical cancer among Romanian women. Persistent infection with high-risk HPV types, particularly HPV 16 and 18, has been confirmed as a primary driver of CIN and cervical cancer progression.

## 1. Introduction

Cervical intraepithelial neoplasia (CIN) refers to a premalignant condition characterized by the abnormal transformation of cervical epithelial cells on the cervix’s surface, located at the lower portion of the uterus [1]. CIN is a significant precursor of cervical cancer, with the potential to progress to invasive forms if not timely diagnosed and appropriately managed [2]. Therefore, understanding and addressing its risk factors are critical for cervical cancer prevention.

Among the various risk factors contributing to CIN, human papillomavirus (HPV) infection is the most significant [3].

Human Papillomavirus (HPV) is a sexually transmitted virus with approximately 200 identified subtypes, each differentiated by their genomic sequences [4]. Around 40 of these types infect the mucosal epithelium and are classified into two groups: low-risk and high-risk, based on their oncogenic potential. The International Agency for Research on Cancer (IARC) has classified 12 high-risk HPV types as Group 1 carcinogens, which include HPV types 16, 18, 31, 33, 35, 39, 45, 51, 52, 56, 58, and 59 [5]. Among these, HPV 16 and 18 are most commonly associated with cervical cancer [6,7,8,9]. However, emerging evidence has highlighted the role of other types, such as HPV 51, in contributing to CIN [10]. It is important to note that current HPV vaccines do not protect against HPV 51.

In addition to HPV infection, several other factors may influence the development of CIN. Lifestyle choices, including smoking, obesity, contraceptive use, and the presence of vaginal infections, have been implicated in CIN progression [11]. These factors can create a microenvironment conducive to HPV persistence and cellular transformation. Given the strong association between HPV infection and CIN, early detection and intervention are essential to halt the progression of cervical cancer. This underscores the importance of comprehensive public health strategies, including HPV vaccination, regular cervical screening, and effective management of associated risk factors [12,13,14,15].

Screening for cervical intraepithelial neoplasia (CIN) and cervical cancer is the cornerstone of preventive healthcare, primarily relying on the Papanicolaou (Pap) smear test and HPV testing [16]. These diagnostic tools play a vital role in the early detection and management of CIN and significantly reduce the risk of progression to invasive cervical cancer. The Pap smear test examines cervical cells for abnormalities, following the Bethesda classification system. This system categorizes findings as normal, atypical squamous cells of undetermined significance (ASCUS), low-grade squamous intraepithelial lesions (LSIL), or high-grade squamous intraepithelial lesions (HSIL) [17]. Abnormal Pap smear results typically warrant further diagnostic investigations, such as colposcopy or biopsy, to confirm the presence and grade of CIN.

CIN is classified into three grades based on the extent of abnormal cell growth within the cervical epithelium [18]:

- CIN 1 (Mild Dysplasia): Involves the lower one-third of the cervical epithelium;
- CIN 2 (Moderate Dysplasia): Affects the lower two-thirds of the cervical lining;
- CIN 3 (Severe Dysplasia/CIS): Involves the full thickness of the cervical epithelium and is also known as carcinoma in situ (CIS), the precursor of invasive cancer.

Histopathological evaluation through biopsy remains the gold standard for confirming CIN and determining its grade [19]. Complementary diagnostic methods, such as HPV DNA testing, enhance screening accuracy by identifying high-risk HPV types associated with CIN and cervical cancer. HPV testing is beneficial for triaging patients with equivocal Pap smear results, such as ASCUS [20,21].

This paper delves into the diagnostic methodologies employed for CIN, emphasizing their role in the early detection and prevention of cervical cancer. By identifying and addressing CIN at its earliest stages, these diagnostic tools contribute to reducing the burden of cervical cancer and improving patient outcomes.

## 2. Materials and Methods

The research study initially included 1049 patients aged 21 to 65 with cervical lesions, enrolled between 2021 and 2024. Several exclusion criteria were applied to refine the study cohort: patients with incomplete cervical investigations, those who failed to attend follow-up appointments, and cases with ASCUS (atypical squamous cells of undetermined significance) or LSIL (low-grade squamous intraepithelial lesion) cytology at the first collection that reverted to NILM (negative for intraepithelial lesion or malignancy) following local medication therapy were excluded. Additionally, young women with spontaneous regression of cervical lesions were excluded. After applying these criteria, the final study cohort comprised 94 patients.

The research was conducted in full compliance with the scientific research regulations established by European and Romanian legislation. Ethics approval was obtained from the local Ethics Committee of the Alco San Medical Center in Bucharest, Romania.

### 2.1. Collection of Clinical Data

The cohort of patients with cervical intraepithelial neoplasia (CIN) was evaluated based on several characteristics, including age, residence (rural or urban), marital status (married, unmarried, or cohabiting), and education level (primary, secondary, or higher education). Additional factors analyzed included living and working conditions, behaviors, family medical history, and obstetrical history (pregnancies, births, and abortions). Personal medical and surgical histories, colpocervical infections caused by various microbial agents (viruses, bacteria, parasites, and fungi), and any medications or treatments received were also considered. Symptomatology, such as leukorrhea, vaginal bleeding, pain, genital burning sensation, local itching, discomfort at rest or during sexual intercourse, and findings from gynecological examinations, were assessed as part of the study.

### 2.2. Collection of Data from Specific Cervical Investigations

The specific cervical investigations performed on the cohort of patients included the following:

- cervicovaginal secretion examination: this involved fungal, bacteriological, and parasitic assessments, along with antibiotic sensitivity testing;
- cervicovaginal cytological examination: conducted using the Papanicolaou/Bethesda system;
- virological examination: included testing for HPV, with HPV genotyping to identify specific viral strains;
- colposcopic examination: performed to visually inspect the cervix for abnormalities;
- histopathological examination: tissue fragments obtained by exocervical biopsy were analyzed for definitive diagnosis.

All these investigations, combined with a detailed medical history and clinical genital examination, led to a positive diagnosis of CIN.

The collection of cervicovaginal secretions required visualization of the cervix to identify macroscopic lesions and locate the squamocolumnar junction. Samples were collected using a cervical brush with longer bristles in the center and shorter bristles on the sides. The brush was rotated clockwise to simultaneously gather cells from the endocervical canal and the exocervix.

Microbiological examination of vaginal secretions was conducted using standard laboratory techniques to identify specific microorganisms. Antibiotic sensitivity testing was performed following the widely accepted Kirby-Bauer disk diffusion method. For fungal and parasitic assessments, specific culture media and microscopic techniques, including wet mount preparations and specialized stains, were employed to ensure accurate pathogen identification.

Vaginal secretion collection was performed outside the menstrual period to avoid blood contamination. Patients were instructed to abstain from sexual intercourse, avoid using intravaginal tampons or local contraceptives, and refrain from vaginal douching for 24–48 h before collection to ensure sufficient cellular desquamation. For patients with colpocervicitis, treatment was recommended during prior visits to resolve inflammation before proceeding with cervicovaginal secretion collection.

For cervicovaginal cytological examination, similar precautions were emphasized. Patients were advised to abstain from sexual intercourse, avoid intravaginal tampons or contraceptives, and refrain from vaginal douching or treatment for 24–48 h before sample collection. Additionally, no vaginal examinations were performed on the morning of sample collection. Cytological samples were obtained from the exocervix, squamocolumnar junction, and endocervix to ensure a comprehensive evaluation.

To ensure the reliability and reproducibility of our microbiological findings, stringent standardization protocols were meticulously implemented throughout the study. All microbiological examinations were conducted using methods approved by clinical laboratory standards, ensuring consistent sample handling, processing, and analysis. Samples were collected under sterile conditions by trained personnel using single-use sterile equipment to minimize the risk of contamination. Each type of microbial culture was incubated under conditions optimized for the growth requirements of a specific organism, and pathogen identification was confirmed via biochemical tests, which are essential for accurate detection.

In the course of our microbiological examinations, we followed stringent protocols designed to prevent contamination risks, which often pose significant challenges in microbiological studies. Our adherence to rigorous aseptic techniques, use of sterilized equipment, and implementation of strict sample handling procedures effectively mitigated potential contamination. We continuously monitored these processes to ensure their effectiveness and made the necessary adjustments to maintain high standards of quality. As a result, no contamination issues were encountered, thereby ensuring the integrity and reliability of the data. The absence of contamination underscores the effectiveness of our preventive measures and supports the validity of our study findings.

The results were interpreted based on the Bethesda classification, which is parallel to the Papanicolaou system. The cervicovaginal cytological examination revealed epithelial cell abnormalities, including LSIL (low-grade squamous intraepithelial lesion), HSIL (high-grade squamous intraepithelial lesion), ASCH (atypical squamous cells, cannot exclude HSIL), and ASCUS (atypical squamous cells of undetermined significance).

HPV detection and genotyping were conducted using real-time PCR. HPV DNA was extracted from cervical samples using the QIAamp DNA Mini Kit (Qiagen, Hilden, Germany) following the manufacturer’s protocol. DNA concentration and purity were assessed using a NanoDrop™ 2000 Spectrophotometer (Thermo Fisher Scientific, Waltham, MA, USA) to ensure suitability for downstream analysis.

For genotyping, we employed the Anyplex™ II HPV28 Detection Kit (Seegene, Seoul, South Korea), which enables the simultaneous detection and differentiation of 28 high-risk and low-risk HPV genotypes using multiplex real-time PCR technology. PCR amplification was performed using a CFX96 Real-Time PCR System (Bio-Rad, Hercules, CA, USA) under the following cycling conditions:

- Initial Denaturation: 95 °C for 15 min;
- Amplification Cycles: 45 cycles of denaturation at 95 °C for 30 s, annealing at 60 °C for 90 s, and extension at 72 °C for 30 s;
- Final Extension: 72 °C for 10 min.

The primers used targeted the L1 region of the HPV genome, ensuring high specificity for viral DNA. Positive and negative controls provided by the manufacturer were included in each PCR run to validate the assay performance and rule out contamination.

HPV genotypes were identified using Seegene Viewer Software Version: 1.0. which automatically interpreted multiplex PCR results. To ensure accuracy and reproducibility, the results were independently verified by two experienced laboratory technicians.

This standardized protocol ensured reliable detection and differentiation of HPV genotypes, enabling a robust analysis of their association with cervical lesion severity.

The high-risk HPV genotypes identified included 16, 18, 31, 33, 35, 39, 45, 51, 52, 53, 56, 58, 59, 66, and 68. Consistent with national recommendations in Romania, HPV testing was primarily performed on patients older than 30 years, where such testing is strongly advised. In Romania, HPV screening is advised for women aged 30 to 64 years, a crucial demographic at higher risk for cervical abnormalities. National guidelines state that women within this age range are entitled to a free HPV test once every five years [22]. Although testing was also recommended for younger patients, it was not systematically performed, likely due to financial constraints.

Colposcopic examination revealed atypical transformation zones (TA I and TA II) at the squamocolumnar junction alongside areas of normal epithelium. The assessment focused on visualizing the squamocolumnar junction, evaluating the entire lesion area, and examining the exocervical vasculature in detail.

The French classification system for atypical transformation (TA I and TA II) was employed and correlated with the Reid Colposcopic Index for accuracy to quantify findings related to cervical intraepithelial neoplasia (CIN).

Histopathological examination confirmed the presence of atypical cells, leading to the diagnosis of various grades of cervical intraepithelial neoplasia: CIN 1 (including lesions associated with HPV infection), CIN 2, and CIN 3.

Histopathological results were compared with cytological and colposcopic findings to assess concordance and potential overestimation or underestimation of cases.

The cohort of patients met the following inclusion criteria:

- sexually active patients;
- patients seeking genital consultations without symptoms or with genital symptoms such as leukorrhea or bleeding but with one of the following conditions:
  - patients with macroscopic cervicovaginal lesions;
  - patients with colpocervical infections caused by various microbial agents (viruses, bacteria, parasites, fungi);
  - patients with obstetrical (births, abortions) and gynecological (medical, surgical) history;
- patients without HPV vaccination;
- patients CIN positive based on retrospective histopathological examination;
- Exclusion criteria:
- sexually inactive patients;
- sexually active patients with one of the following conditions:
  - patients with incomplete monitoring and investigation;
  - young patients under surveillance for lesions;
  - patients with initial ASCUS or LSIL cytology who later became NILM;
  - patients who did not attend follow-up appointments or sought care at other specialized centers;
- patients who received HPV vaccination.

### 2.3. Statistical Analysis

Statistical analyses were conducted to evaluate the association between microbial agents, HPV genotypes, and cervical lesion severity. Chi-square and Fisher’s exact tests were utilized, with a significance threshold set at *p* < 0.05. All statistical computations were performed using SPSS Statistics software, version 26 (IBM Corp., Armonk, NY, USA). We set a significance threshold at *p* < 0.05 for all tests. This threshold was chosen because it is widely accepted in biomedical research as it minimizes the likelihood of Type I errors while maintaining adequate power to detect true associations within our data. This approach balances the risk of drawing false-positive conclusions (Type I errors) with the risk of overlooking true associations (Type II errors), thereby enhancing the validity and reliability of our findings. The results were stratified by lesion grade, enabling the identification of pathogen-specific and genotype-specific trends associated with the progression of cervical dysplasia.

## 3. Results

### 3.1. Presentation of the Study Group

**Figure 1:**
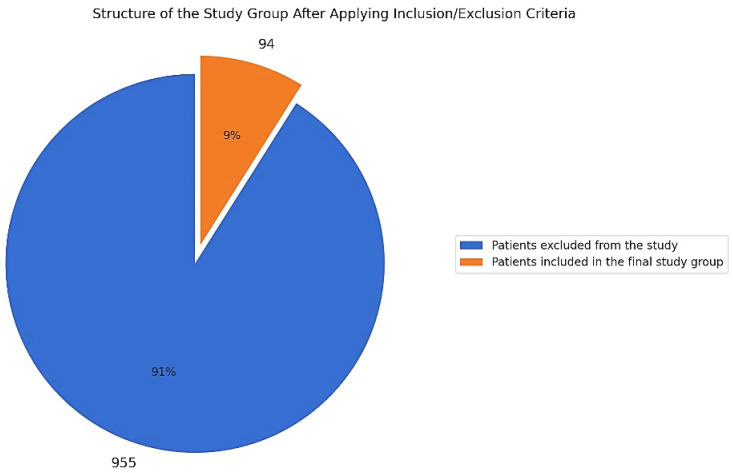
Distribution of cases in the study cohort.

The research study initially included 1049 patients with cervical lesions, aged 21 to 65 years, enrolled between 2020 and 2024. After applying the exclusion criteria, the final study cohort consisted of 94 patients (Figure 1).

The patients were categorized into five age groups: 21–30 years, 31–40 years, 41–50 years, 51–60 years, and over 61 years (Table 1). The mean age of the cohort was 39.54 years (±11.02).

Comprehensive patient anamnesis included details regarding the number of births and abortions in their medical history. Cervical trauma sustained during vaginal delivery has been identified as a potential contributor to cervical lesions, which, when combined with infectious agents (viral, bacterial, parasitic, or fungal) and hormonal factors, can progress to cervical dysplasia.

In examining the relationship between CIN and the number of births, the lowest incidence of cervical dysplasia was found among nulliparous patients, while the highest incidence was observed in those with one to three births (Table 2).

The distribution of cases based on abortion history, as shown in Table 3, reveals a main trend. Women with one to three abortions comprised the largest group, representing 53.20% of the cases, followed by those with more than four abortions at 38.30%. Only 8.50% of the cases were observed in women without a history of abortion. These findings indicate a strong relationship between the frequency of abortions and the prevalence of CIN, emphasizing the potential impact of repeated cervical trauma on its development.

### 3.2. Presentation of the Study Group Based on Specific Cervical Investigations

Microbiological examination of vaginal secretions revealed a diverse range of infections, with diagnoses supported by antibiotic sensitivity testing to guide appropriate treatment. Among the 94 patients studied, 46.80% (44 cases) exhibited sterile vaginal secretions, while 53.20% (50 cases) showed infections with one or more microbial agents. This finding indicates a high prevalence of cervicovaginal infections in patients with cervical lesions.

The distribution of cases according to the number of infections is summarized in Table 4. A single microbial infection was identified in 10.65% of the patients, while 34.04% presented with two distinct infections. Notably, 8.51% of patients had infections involving more than three microbial agents, highlighting the complexity of polymicrobial infections in some instances.

Among patients with cervicovaginal infections associated with HPV, a variety of microbial pathogens was identified. The most frequently detected pathogen was *Candida albicans* (28% of cases). Gram-positive cocci (*Streptococcus* spp. and *Staphylococcus* spp.) were the second most common (24%), followed by *Escherichia coli* (16%). *Trichomonas vaginalis* and *Gardnerella vaginalis* were often found together, accounting for 10% and 8% of cases, respectively. Less frequent but clinically significant pathogens included *Mycoplasma genitalium* (4%), *Ureaplasma urealyticum* (4%), and *Chlamydia trachomatis* (6%), which are known to influence HPV infection progression and the female genital tract’s inflammatory response (Table 5).

A detailed analysis was conducted to investigate the association between the type and number of microbial agents and the severity of cervical lesions. Among the 50 patients with microbial infections, *Candida albicans* was predominantly associated with low-grade cervical intraepithelial neoplasia (CIN 1) (*p* = 0.04). In contrast, *Escherichia coli* and *Trichomonas vaginalis* showed a significant correlation with high-grade CIN (CIN 2/3) (*p* < 0.05). Patients with polymicrobial infections involving three or more pathogens were more likely to present with high-grade lesions compared to those with single-pathogen infections or sterile vaginal secretions (*p* = 0.03). While *Gram-positive cocci* (e.g., *Streptococcus* spp. and *Staphylococcus* spp.) were commonly identified, no statistically significant association with lesion severity was observed (Table 6).

The cervicovaginal cytological examination revealed a wide range of epithelial cell abnormalities, ranging from borderline atypical findings to indications of invasive disease (Table 7). The most common finding was high-grade squamous intraepithelial lesion (HSIL), followed by low-grade squamous intraepithelial lesion (LSIL) and atypical squamous cells of undetermined significance (ASCUS). While atypical squamous cells cannot exclude HSIL (ASCH), it was less frequent.

Colposcopic examination, followed by biopsy and histopathological evaluation, was essential to confirm the grade of cervical dysplasia.

High-risk HPV (HR-HPV) genotyping was performed in 72 patients, primarily those over 30 years of age, to identify specific viral strains linked to cervical dysplasia and carcinoma. Genotyping targeted 15 HR-HPV types, including types 16 and 18, which are recognized for their strong oncogenic potential. The results revealed that HPV 16 and 18, the most oncogenic genotypes, were predominantly found in patients under the age of 40 (Table 8).

Further investigation explored the relationship between HPV genotypes and the severity of cervical lesions. Among the 72 patients with HR-HPV infections, HPV 16 and 18 were significantly associated with high-grade CIN (CIN 2/3) and invasive carcinoma (*p* < 0.001). Non-16/18 HR-HPV genotypes (e.g., 31, 33, 45, and 51) were more commonly linked to low-grade CIN (CIN 1) (*p* = 0.02). Co-infection with multiple HPV genotypes was observed in 10% of the cases, with a notable predominance in patients with high-grade lesions (*p* < 0.05) (Table 9).

Patients aged 50–65 years predominantly had infections with high-risk HPV genotypes other than HPV 16 and 18, including HPV 31, 33, 35, 39, 45, 51, 52, 53, 56, 58, 59, 66, and 68. In this age group, only four cases (21.05%) were infected with HPV 16 and 18, while 15 patients (78.95%) had infections with other high-risk HPV genotypes.

The prevalence of high-risk HPV (HR-HPV) infections varied by age group. Infections with HPV 16 and 18 were significantly higher among patients aged 31–40 years (*p* < 0.05), while infections with other high-risk HPV types were significantly more common in patients aged over 50 years (*p* < 0.05).

Colposcopic examinations were performed on patients with abnormal cytological findings to assess the severity of the lesions. During the examination:

- suspicious lesions were identified, with their topographic location, size, and severity recorded;
- biopsies were taken from the most severe lesions as well as from areas of apparently normal epithelium;
- special attention was given to examining the squamocolumnar junction for abnormalities.

Lesions were classified based on atypical transformation zones (grades I and II), considering factors such as lesion contour and color, the presence of de novo neovascularization, and response to iodine staining.

The colposcopic examination was negative for dysplasia in only 4.25% of the cases, highlighting a strong correlation between cervicovaginal infection, cytological findings, and HPV presence (Table 10).

Histopathological analysis of tissue samples obtained through biopsy revealed low-grade cervical intraepithelial neoplasia (CIN 1), high-grade CIN (CIN 2 and CIN 3), and invasive cancer, as illustrated in Table 11.

Cervicovaginal cytology results and histopathological diagnoses were determined through exocervical biopsy. The results are summarized in Table 12.

Histopathological examination confirmed low-grade CIN in 46 patients (48.94%), including 14 cases of ASCUS, 26 cases of LSIL, and 6 cases of HSIL. High-grade CIN was confirmed in 40 patients (42.55%), including 4 cases of ASCUS, 5 cases of LSIL, 28 cases of HSIL, and 3 cases of cells suggestive of squamous neoplasia. Squamous cancer was confirmed in eight cases (8.51%), including five cases detected through cytology suggestive of squamous carcinoma, one case of LSIL, and two cases of HSIL.

Although atypical squamous cells were detected in 18 cases (19.15%), their significance could not be precisely determined, and they were classified as ASCUS or ASCH. Cytological predictions for low- and high-grade cervical intraepithelial neoplasia and squamous carcinoma were consistent with histopathological results in 59 cases (62.77%), underestimated in 8 cases (8.51%), and overestimated in 9 cases (9.57%).

The best predictive accuracy was observed for high-grade CIN, which correlated well with HSIL cytology in 70% of the cases. For low-grade CIN, the correlation with LSIL cytology was 56.52%. Microinvasive carcinoma was suspected based on cervicovaginal cytology in 62.5% of the confirmed cases.

We further analyzed the concordance between the colposcopic findings and histopathological diagnosis established via exocervical biopsy (Table 13).

Histopathological examination confirmed low-grade CIN in 46 patients (48.94%), which included 4 cases negative for dysplasia, 34 cases of grade I atypical transformation, 7 cases of grade II atypical transformation, and 1 case with colposcopic findings suggestive of invasive cancer. High-grade CIN was diagnosed in 40 patients (42.55%), which included 14 cases of grade I atypical transformation, 22 cases of grade II atypical transformation, and 4 cases with colposcopic findings suggestive of invasive cancer. Invasive squamous cancer was confirmed in eight cases (8.51%), including five cases of grade II atypical transformation and three cases suggestive of invasive cancer based on colposcopy.

Analysis of colposcopy as a predictive tool for cervical intraepithelial neoplasia (CIN) and squamous carcinoma revealed concordant results in 59 cases (62.77%), underestimation in 19 cases (20.21%), and overestimation in 12 cases (12.76%).

For low-grade CIN, colposcopy findings correlated well with histopathology in 73.91% of cases involving grade I atypical transformation. For high-grade CIN, the correlation with grade II atypical transformation findings was 55%. Among patients with histologically confirmed squamous carcinoma, the correlation with colposcopy findings was 37.5%.

A comparison of cervicovaginal cytology and colposcopy results against histopathological diagnoses showed that for low-grade CIN, colposcopy outperformed cytology with a 17.39% difference (*p* = 0.02), indicating a statistically significant difference (*p* < 0.05). For high-grade CIN, cervicovaginal cytology showed a 15% advantage over colposcopy (*p* = 0.04, *p* < 0.05). For invasive squamous carcinoma, cervicovaginal cytology had a 25% higher accuracy (*p* = 0.0001, *p* < 0.05).

While concordant results between cytology and colposcopy were observed in 59 (62.76%) of the 94 patients diagnosed with CIN or invasive squamous carcinoma, a significant portion (37.24%) of the final diagnoses relied on additional suspicion raised by one of the diagnostic methods, HPV detection and genotyping, analysis of associated microbial flora, patient age, and medical history, and further investigation of inconclusive cases.

## 4. Discussion

According to data from the European Cancer Information System (ECIS) of the Joint Research Centre, Romania recorded the highest incidence and mortality rates for cervical cancer in the European Union in 2022. The incidence rate in Romania was 32.6 per 100,000 women, which is five times higher than that in Finland, which is 6.4 per 100,000 women. Additionally, Romania’s mortality rate was 16.8 per 100,000 women, eight times greater than Finland’s rate of 2.2 per 100,000 women [23].

This study extends our understanding of the interplay between cervicovaginal infections and HPV-related cervical pathologies. We have meticulously reviewed recent publications, integrating studies that explore similar themes, particularly those conducted in the last five years, to provide a contemporary backdrop that highlights the relevance and urgency of our research.

Our study uniquely quantifies the impact of specific microbial agents, like Candida albicans, on the progression of cervical intraepithelial neoplasia (CIN) within a Romanian cohort. This approach not only fills a significant gap by contributing regional data but also suggests potential for region-specific therapeutic interventions, enhancing our understanding of molecular pathogenesis.

Our findings are discussed in the context of global health trends, particularly focusing on strategies for cervical cancer prevention. We analyze how our insights could influence screening and vaccination strategies in Eastern Europe, thereby drawing connections between localized research and global health objectives.

Our discussion addresses critical gaps identified through our research, proposing future directions, such as the need for longitudinal studies to explore the dynamic changes in the cervicovaginal microbiota over time and its effect on HPV clearance and CIN progression.

The early diagnosis of CIN and its appropriate therapeutic management, combined with HPV vaccination programs, represent the most effective strategies for preventing cervical cancer [24,25,26,27]. Cervical cancer remains the most prevalent genital cancer among women and the second leading cause of cancer-related deaths in our country, following breast cancer [28,29]. A significant contributor to this high disease burden is the low participation rate of women in cervical cytology screening programs. It highlights the urgent need to improve educational outreach and health standards across the population [30,31]. Efforts to reduce risk factors, promote HPV vaccination, and implement regular Papanicolaou cytology screenings for the early detection and monitoring of cervical precursor lesions could significantly lower the incidence of cervical cancer in Romania [31,32,33].

CIN typically progresses to invasive cervical cancer over approximately ten years, with the majority of cases diagnosed around the age of 38 [34]. In our study, statistical analysis revealed a mean patient age of 39.54 years (±11.02), with participants in the final cohort ranging in age from 21 to 65 years.

The diagnosis and monitoring of patients with cervical lesions require a comprehensive approach that incorporates multiple factors. It includes clinical inspection of the cervicovaginal area, bimanual vaginal examination combined with abdominal and rectal palpation, and a series of specific cervicovaginal investigations. These investigations encompass microbiological examination of cervicovaginal secretions, HPV detection and genotyping, cervicovaginal cytology, colposcopy, and biopsy with histopathological analysis. Additionally, patient demographics, including age, family medical history, and obstetric and medical history, are essential for a thorough evaluation.

Microbiological analysis of cervicovaginal smears in this study revealed that 53.20% of the patients had at least one genital infection. These infections can disrupt the cervicovaginal flora, increasing susceptibility to other carcinogenic factors and contributing to dysplastic lesions [35,36]. Although vaginal infections are not direct causes of intraepithelial lesions, their presence compromises cervical mucosal defenses, thereby facilitating the progression of these lesions.

Recent studies have underscored the significant impact of vaginal microbiota and cervicovaginal infections on the development of CIN. An imbalanced vaginal microbiome, marked by a reduction in protective *Lactobacillus* species and overgrowth of pathogenic organisms, often results in altered vaginal pH levels, thereby increasing the risk of cervical abnormalities [37,38]. Bacterial vaginosis, a polymicrobial vaginal dysbiosis characterized by reduced *Lactobacillus* levels, is a common contributor to these changes [39].

Recent research has identified distinct bacterial signatures that distinguish healthy vaginal microbiota from those associated with infections [40]. Both bacterial vaginosis and *Chlamydia trachomatis* infections have been linked to an elevated risk of HPV infection, with bacterial vaginosis being particularly associated with a higher likelihood of CIN development [41]. However, co-infection with *Candida albicans* has not been shown to enhance the carcinogenic effects of HPV on the cervix [41,42].

Our findings indicate an association between microbial infections and CIN severity. *Candida albicans, Escherichia coli*, and *Trichomonas vaginalis* have been identified as the main pathogens associated with the advancement of cervical lesions. Polymicrobial infections are correlated with high-grade CIN, suggesting that a disrupted cervicovaginal microbial environment may exacerbate lesion severity and impede the immune response. These observations align with emerging evidence highlighting the multifaceted role of the cervicovaginal microbial spectrum in CIN pathogenesis.

The cervicovaginal microbial spectrum plays a critical role in the pathogenesis and progression of CIN, often acting synergistically with high-risk HPV infections [36]. A balanced vaginal microbiome, dominated by *Lactobacillus* species, provides a protective barrier by producing lactic acid and hydrogen peroxide, maintaining an acidic vaginal pH, and preventing colonization by pathogenic microorganisms [43,44]. In contrast, microbial dysbiosis—characterized by an overgrowth of opportunistic pathogens such as *Candida albicans*, *Escherichia coli*, and *Trichomonas vaginalis*—has been associated with HPV persistence, immune evasion, and chronic inflammatory responses [45,46]. These pathogens can disrupt epithelial integrity, facilitate viral entry, and impair local immune defenses, creating an environment conducive to viral persistence and lesion progression [47,48].

In our study, *Candida albicans* was predominantly associated with low-grade CIN (CIN 1), suggesting its correlation with early dysplastic changes. Conversely, *Escherichia coli* and *Trichomonas vaginalis* showed stronger associations with high-grade CIN (CIN 2/3), indicating their potential roles in driving disease progression. Furthermore, polymicrobial infections were significantly more prevalent in high-grade lesions, suggesting a synergistic effect that may amplify inflammatory responses, disrupt epithelial barriers, and accelerate CIN progression.

The mechanisms underlying these associations are likely to be multifactorial. Chronic inflammation induced by microbial agents can impair mucosal immunity, increase cytokine production, and promote cellular damage, all of which facilitate HPV persistence and viral integration into host cells [49,50]. Pathogens such as *Escherichia coli* and *Trichomonas vaginalis* have also demonstrated direct interactions with epithelial cells, further contributing to tissue damage and dysplasia [51,52,53].

While our findings provide valuable insights into the complex interactions between microbial agents and CIN progression, we recognize the challenges in isolating the specific contributions of individual pathogens, especially in cases of polymicrobial infections. Future studies utilizing advanced microbial profiling technologies, including next-generation sequencing (NGS) and metagenomic analysis, are essential to delineate these relationships more precisely.

HPV genotyping, on the other hand, plays a pivotal role in understanding CIN progression and its transition to invasive cervical cancer [54]. Persistent infection with high-risk HPV genotypes, particularly HPV 16 and 18, is well established as the primary driver of cervical carcinogenesis [55]. Genotyping enables the precise identification of these high-risk strains, offering valuable insights into their differential oncogenic potential and their association with lesion severity [56]. Evidence consistently indicates that HPV 16 and 18 are disproportionately linked with high-grade CIN (CIN 2/3) and invasive carcinoma, whereas other high-risk genotypes, such as HPV 31, 33, 45, and 51, exhibit a stronger association with low-grade lesions (CIN 1) [57,58,59,60,61].

These genotype-specific differences highlight the importance of HPV genotyping in risk stratification, enabling clinicians to prioritize high-risk individuals for more intensive surveillance and early therapeutic interventions [62,63]. Furthermore, HPV genotyping facilitates post-vaccination surveillance, allowing monitoring of genotype prevalence in vaccinated and unvaccinated populations and assessing vaccine efficacy over time [64,65,66].

Our analysis of HPV genotyping confirmed the predominant oncogenic potential of HPV 16 and 18, which were strongly associated with high-grade CIN and invasive carcinoma. Additionally, we observed a higher prevalence of non-16/18 high-risk HPV genotypes in cases of low-grade CIN, indicating that these genotypes may follow distinct and potentially less aggressive pathogenic pathways. This variation in pathogenicity highlights the importance of detailed HPV genotyping to understand lesion progression and develop tailored patient management strategies.

In HPV-positive women, the vaginal microbiota often exhibits increased microbial diversity and reduced relative abundance of *Lactobacillus* species, accompanied by elevated vaginal pH levels [67]. These changes in the microbiome can create an environment conducive to cervical dysplasia progression.

Co-infection with *Chlamydia trachomatis* is particularly significant. This pathogen exacerbates HPV-induced cellular changes by inhibiting apoptosis and facilitating the integration of HPV DNA into host cells [68,69,70]. Among its serotypes, *Chlamydia trachomatis* serotype G is strongly associated with severe dysplastic lesions and cervical squamous carcinoma [71,72]. Additionally, studies have shown that smokers co-infected with HPV 16 and *Chlamydia trachomatis* have a heightened risk of developing cervical cancer [73,74,75].

The influence of microbial agents like *Candida albicans* or *Chlamydia trachomatis* alongside HPV underscores the importance of a comprehensive approach to screening and treatment. This approach should include targeted antimicrobial therapy, HPV genotyping, and addressing modifiable risk factors, such as smoking.

Cervicovaginal infections contribute to a microenvironment favorable for HPV persistence and progression to CIN through several mechanisms:

- cause chronic inflammation, which facilitates HPV infection, replication, and integration into host cells;
- disrupt the vaginal microbiome, potentially weakening local immune defenses and reducing the body’s ability to clear HPV;
- serve as cofactors in the progression of HPV infection to CIN.

The Papanicolaou cervical cytology test plays a critical role in the early detection of cellular abnormalities in most cases of cervical intraepithelial neoplasia or carcinoma in situ, enabling optimal therapeutic management [76]. With a specificity of approximately 98%, the Papanicolau test is highly reliable for identifying true positives. However, its sensitivity is low and can vary, which underscores the necessity for periodic screening [77,78]. Recommendations for screening suggest that women aged 21 to 65 should undergo Pap testing every three years, even if they do not experience any symptoms [79,80,81]. False-negative results often occur due to improper sampling techniques or failure to identify abnormal cervical cells during cytological analysis [82]. To enhance early detection and reduce the incidence of cervical cancer, it is vital to ensure high-quality sampling and strictly adhere to screening intervals.

In this study, atypical squamous cells were detected in 18 cases (19.15%); however, their exact significance could not be determined, and they were classified as ASC (ASCUS and ASCH). The cytological predictions for low- and high-grade intraepithelial neoplasia and squamous carcinoma correlated with the histopathological results in 59 cases (62.77%). In eight cases (8.51%), cytology underestimated the findings, while in nine cases (9.57%), it overestimated the severity. The highest diagnostic accuracy was observed for high-grade CIN, where cytological HSIL findings correlated with histopathology in 70% of the cases. In low-grade CIN, the correlation with LSIL cytology was 56.52%. Microinvasive carcinoma was suspected based on cervicovaginal cytology in 62.5% of confirmed cases.

These findings are consistent with the literature, which supports the high sensitivity and specificity of cervicovaginal cytology for detecting CIN and early-stage cervical cancer. However, its specificity for predicting dysplasia severity remains limited. Cervicovaginal cytology is particularly valuable for population screening, but a significant percentage of cervical lesions may yield false-negative results [83,84], a range that aligns with the findings of this study. Combining HPV testing with cytology enhances the sensitivity of the Papanicolaou test for detecting high-grade lesions from 50–85% to 100% [85,86]. Some researchers advocate the use of HPV testing alone as a primary screening method for cervical cancer prevention [87,88]. HPV testing is more sensitive than the Papanicolaou test and facilitates the early detection of high-grade neoplasia; however, it has lower specificity, particularly in younger women [81,89,90].

Genital HPV infection is the most common sexually transmitted infection, with an 80% lifetime risk of acquiring genital HPV, and the highest incidence occurring in women under 25 years of age [91,92]. The primary cause of cervical lesions is persistent infection with HPV. HPV types 16, 18, 45, and 31 are associated with high-grade CIN, which has a significant malignant potential [26,93,94]. High-risk HPV types are the primary etiological factors for pre-invasive and invasive cervical lesions. HPV-16, in particular, is present in 40–70% of invasive cancers worldwide [7,95,96]. Although HPV-18 is less prevalent, it accounts for 12% of squamous cell carcinoma and 37% of adenocarcinoma of the cervix worldwide [97,98,99].

Globally, in countries with low cervical cancer incidence, chronic HPV infection prevalence is 5–10% [3]. In countries with high cervical cancer incidence, persistent HPV infection rates range from 10% to 20% [3]. Vaccination against HPV prevents infection by high-risk oncogenic strains, reducing the risk of cervical dysplasia and cancer [100].

Cytological screening using the Papanicolaou test or Bethesda system, along with viral genotyping to detect high-risk HPV DNA, can identify high-grade lesions and cervical cancer [101,102,103,104]. High-grade lesions (HSIL, ASC-H) carry a high risk of progression to invasive cancer, and to prevent further progression, it is essential to monitor cytological and viral changes, with the possibility of subsequent histological evaluation through cervical biopsy [105,106,107].

For ASCUS cytological results, HPV testing is recommended due to the high sensitivity of this test for detecting high-grade squamous intraepithelial neoplasia (HSIL). Moreover, HPV testing offers a favorable cost-benefit ratio compared to repeated cytology or colposcopy, making it an effective tool for risk stratification and early detection of significant cervical lesions [108,109].

In this study, all cases with ASCUS cytological results were HPV-positive, likely due to the high sensitivity of the real-time PCR method used for HPV detection and the selection of a high-risk cohort with preexisting cervical lesions. This finding reflects the strong association between ASCUS and HPV infection in populations with elevated HPV prevalence, particularly in high-risk genotypes. The strict inclusion criteria, focusing on cases with confirmed or suspected cervical dysplasia, may have further contributed to the 100% HPV positivity rate observed in this group.

HPV detection and genotyping can reveal high-grade squamous intraepithelial neoplasia, even when colposcopy shows minimal transformation zone changes, which may histopathologically suggest CIN 1 [110,111]. Thorough colposcopic examination of mild transformation zone changes is crucial, and in uncertain cases, HPV testing can enhance both the sensitivity and specificity in detecting high-grade lesions [112].

Randomized clinical trials have demonstrated that a negative cytology result combined with positive HPV testing for types 16 or 18 corresponds to a high risk of CIN III and indicates immediate colposcopy [113,114,115]. Screening can be performed exclusively through HPV DNA testing [116,117], with patients who test positive for HPV triaged through cytology or genotyping. Patients with ASCUS cytology or positive results for HPV-16 and HPV-18 should undergo colposcopy [75,106]. HPV-16 and 18 are the most common and virulent strains, increasing the risk of cervical carcinoma in patients with persistent infections compared to women infected with other high-risk HPV strains [118]. Identifying patients at the highest risk of developing cervical neoplasia through HPV genotyping is a vital tool for risk assessment.

In cases of suspicious cytology, follow-up colposcopy and biopsy are essential for optimizing early detection and prevention of cervical lesions and cancer. This study identified a higher prevalence of HPV-16 and 18 infections in patients aged 31–40 years (68.96%) compared to those over 51 years (78.95%), who more frequently had other high-risk HPV genotypes (31, 33, 35, 39, 45, 51, 52, 53, 56, 58, 59, 66, 68).

Colposcopy is a vital tool in the management of CIN and evaluation of abnormal cytology results. It allows for direct visualization and biopsy of the cervix, enabling accurate diagnosis and staging of CIN, including high-grade lesions and squamous carcinoma. The sensitivity of colposcopy for detecting high-grade CIN has significantly improved over the years. It has increased from 39% to 45.8% and even up to 85.5% in some studies, highlighting its growing importance in detecting cervical lesions [119,120,121]. This progress underscores the need for continued cytological and colposcopic monitoring for the accurate assessment and management of cervical abnormalities.

This study had a 62.77% concordance between the colposcopic findings and histopathological results. This indicates that in most cases, the colposcopic diagnosis aligned with the histological findings from biopsies. However, there were discrepancies: in 19 cases (20.21%), colposcopy underestimated the severity of lesions, while in 12 cases (12.76%), it overestimated the severity, which is typical when evaluating complex or mixed lesions. These discrepancies emphasize the need for complementary histopathological examination to confirm colposcopic findings.

In low-grade CIN cases, the highest correlation was observed with grade I atypical transformation (TA I), with 73.91% concordance. This finding suggests that colposcopy is highly effective in detecting low-grade lesions, particularly when the transformation zone exhibits mild atypia. For high-grade CIN, the correlation was lower, at 55%, with grade II atypical transformation (TA II). This finding indicates that high-grade lesions may present with more subtle or complex features, making them harder to assess accurately through colposcopy alone.

In patients with histologically confirmed squamous carcinoma, the colposcopic findings correlated with the histological diagnosis in only 37.5% of cases, suggesting that colposcopy may not be as reliable for detecting invasive cancer, particularly in cases of multiple or mixed cervical lesions where a normal epithelium may hide primary features. While colposcopy is a valuable diagnostic tool, it has limitations, mainly when dealing with multiple or mixed cervical lesions, where features may be challenging to identify or distinguish. These cases may require a more comprehensive approach that includes additional biopsies. Colposcopy-guided biopsy typically detects 60–70% of high-grade lesions. However, multiple biopsies may be needed for accurate diagnosis, including areas that appear normal under visual inspection but may contain precancerous or cancerous cells [122,123].

Colposcopy plays an essential role in identifying features of cervical dysplastic lesions, allowing for the identification of critical features such as lesion polymorphism and intensely acidophilic lesions seen endocervical. These findings can indicate CIN progression or suggest severe dysplasia or carcinoma in situ. Colposcopy also aids in identifying the association between dysplastic lesions and HPV infection. The development of irregular, coarse, corkscrew-like vessels is a strong indicator of CIN 3, carcinoma in situ, and invasive cervical cancer [124,125]. These patterns evolve from regular fine vascular networks in healthy cervical tissues.

Biopsied tissue fragments are subjected to histopathological examination to confirm the presence of atypical cells characteristic of CIN, particularly in those with a high risk of progression.

Histopathological biopsy remains the gold standard for diagnosis despite its potential discomfort to patients and anxiety associated with waiting for results [126,127,128]. A cervical lesion diagnosis is most accurate when HPV testing, cytology, colposcopy, and histopathology are concordant. A biopsy is essential for providing deeper insights into the type and grade of the lesion and for guiding treatment. It is important to note that in some cases, progression from severe dysplasia to cervical biopsy can occur rapidly, even within 2–3 years after normal cytology, underling the importance of early diagnosis and treatment to prevent disease progression [129].

The concordance between HPV testing, cytology, colposcopy, and histopathology ensures the most accurate and comprehensive diagnosis of cervical lesions. Comparing the concordance levels of cervicovaginal cytology and colposcopy with histopathological diagnoses from cervical biopsies revealed a statistically significant 17.39% advantage in favor of colposcopy for low-grade intraepithelial neoplasia (*p* = 0.02). For high-grade intraepithelial neoplasia, there was a 15% difference in favor of cervicovaginal cytology (*p* = 0.04). For squamous cervical cancer, a 25% difference in favor of cervicovaginal cytology was observed (*p* = 0.0001).

Although positive concordance between the two tests was observed in 59 cases (62.76% of the 94 patients diagnosed with CIN or invasive squamous cancer), a significant proportion (37.24%) of the final diagnosis relied on additional findings from HPV detection and genotyping, the identification of associated microbial flora, patient age and history, and investigations into inconclusive cases.

From a statistical perspective, cytology, high-risk HPV infection, colposcopy, and histopathology were significantly correlated with patient age, forming a diagnostic and prognostic scale (χ^2^ test, *p* < 0.05).

The integration of cytology, colposcopy, HPV testing, and histopathology provides a solid background for diagnosing and managing cervical lesions. While each diagnostic tool has strong points, colposcopy excels in detecting low-grade lesions, cytology is more effective for identifying high-grade lesions and invasive carcinoma, and histopathology remains indispensable for definitive diagnosis. Combining these methods improves the overall accuracy and ensures timely detection, especially for high-risk patients, reducing the likelihood of missed diagnoses and late-stage cancer progression.

## 5. Suggestions for Future Research

Future research should prioritize multi-center studies with larger, more diverse cohorts to enhance the external validity of the findings. Advanced molecular diagnostics, such as next-generation sequencing (NGS) and transcriptomic profiling, should be integrated to explore the genetic and epigenetic mechanisms underlying CIN progression. Longitudinal studies are needed to elucidate the natural history of CIN and the impact of persistent HPV infection over time. Further exploration of the vaginal microbiome’s role in cervical dysplasia development could identify potential microbial biomarkers for disease progression. Evaluating the effectiveness of current HPV vaccines against non-covered strains, particularly HPV 51, is critical for informing future vaccination strategies. Additionally, cost-effectiveness analyses of integrated screening methods combining cytology, HPV testing, and genotyping should guide protocol optimization. Investigating host immune responses and their interactions with microbial co-pathogens may provide insights into novel therapeutic targets for CIN prevention and management.

## 6. Limitation of the Study

The present study has several limitations that warrant consideration. Firstly, the small sample size of 94 patients limits the generalizability of the findings to larger populations. Secondly, the retrospective component of the study may introduce selection and information biases due to incomplete or inconsistent patient records. Thirdly, the study was conducted in a single-center setting, which may not fully represent diverse populations or healthcare systems. Additionally, HPV genotyping was restricted to patients over the age of 30 years, potentially excluding relevant insights into HPV strain distribution in younger women. Finally, excluding patients with spontaneous lesion regression or those lost to follow-up could introduce selection bias, particularly in understanding the natural history of low-grade lesions.

## 7. Conclusions

This study emphasizes the mutual effect of cervicovaginal infections and HPV infection in increasing the risk of developing CIN and cervical cancer among Romanian women. Persistent infection with high-risk HPV types, particularly HPV 16 and 18, was confirmed as a primary driver of CIN and cervical cancer progression.

Effective public health measures, such as HPV vaccination and early detection and treatment of genital infections, are important tools for reducing the morbidity and mortality associated with cervical cancer in this population.

Maintaining microbial health is a fundamental component of cervical cancer prevention strategies, highlighting the importance of addressing both viral and microbial factors.

## Figures and Tables

**Table 1:** Distribution of cases by patient age.

Age Group	Number of Cases	Percentage (%)
21–30	22	23.40
31–40	29	30.85
41–50	24	25.53
51–60	15	15.96
61–65	4	4.26
Total	94	100

**Table 2:** Distribution of cases by number of births.

Number of Births	Number of Cases	Percentage (%)
0	12	12.77
1–3	48	51.06
>4	34	36.17
Total	94	100

**Table 3:** Distribution of cases by number of abortions.

Number of Abortions	Number of Cases	Percentage (%)
0	8	8.50
1–3	50	53.20
>4	36	38.30
Total	94	100

**Table 4:** Distribution of patients by number of microbial infections.

Number of Infections	Number of Cases	Percentage (%)
0	44	46.80
1	10	10.65
2	32	34.04
>3	8	8.51
Total	94	100

**Table 5:** Distribution of patients by associated microbial infection.

Microbial Pathogen	Number of Cases	Percentage (%)
*Candida albicans*	14	28
Gram-positive cocci (*Streptococcus* spp., *Staphylococcus* spp.)	12	24
*Escherichia coli*	8	16
*Trichomonas vaginalis*	5	10
*Gardnerella vaginalis*	4	8
*Mycoplasma genitalium*	2	4
*Ureaplasma urealyticum*	2	4
*Chlamydia trachomatis*	3	6
Total	50	100

**Table 6:** Association Between Microbial Agents and Cervical Lesion Severity.

Microbial Agent	CIN 1 (Low-Grade CIN)		CIN 2/3 (High-Grade CIN)		Invasive Carcinoma		Total Cases
	No.	%	No.	%	No.	%	
*Candida albicans*	10	71.43	4	28.57	0	0	14
*Gram-positive cocci*	8	66.67	4	33.33	0	0	12
*Escherichia coli*	3	37.5	5	62.5	0	0	8
*Trichomonas vaginalis*	2	40	3	60	0	0	5
*Gardnerella vaginalis*	3	75	1	25	0	0	4
*Mycoplasma genitalium*	1	50	1	50	0	0	2
*Ureaplasma urealyticum*	1	50	1	50	0	0	2
*Chlamydia trachomatis*	2	66.67	1	33.33	0	0	3
Polymicrobial Infections	12	37.5	15	46.88	5	15.62	32

CIN = cervical intraepithelial neoplasia.

**Table 7:** Distribution of cases by cytological examination.

Cytological Examination	Number of Cases	Percentage (%)
ASCUS	10	10.64
ASCH	8	8.51
LSIL	32	34.04
HSIL	36	38.30
Cells Suggestive of Carcinoma	8	8.51
Total	94	100

ASCUS = atypical squamous cells of undetermined significance, ASCH = atypical squamous cells, LSIL = low-grade squamous intraepithelial lesion, HSIL = high-grade squamous intraepithelial lesion.

**Table 8:** Distribution of patients by High-risk Human Papillomavirus genotyping and age group.

HPV Type	HPV 16	HPV 18	Other HR-HPV	Total	Percentage (%)
31–40 years age group	12	9	8	29	30.85
41–50 years age group	7	5	12	24	25.53
51–60 years age group	2	2	11	15	15.96
61–65 years age group	0	0	4	4	4.26
Total	21	16	35	72	100

HPV = Human Papillomavirus, HR-HPV = High-risk Human Papillomavirus.

**Table 9:** Distribution of High-risk Human Papillomavirus Genotypes and Cervical Lesion Severity.

HPV Genotype	CIN 1 (Low-Grade CIN)		CIN 2/3 (High-Grade CIN)		Invasive Carcinoma		Total Cases
	No.	%	No.	%	No.	%	
HPV 16	12	29.27	25	60.98	4	9.76	41
HPV 18	9	30.00	18	60.00	3	10.00	30
Non-16/18 HR-HPV	20	54.05	15	40.54	2	5.41	37
Multiple HPV Genotypes	5	35.71	8	57.14	1	7.14	14

HPV = Human Papillomavirus, HR-HPV = High-risk Human Papillomavirus.

**Table 10:** Distribution of cases by colposcopic findings.

Colposcopic Examination	Number of Cases	Percentage (%)
Negative for Dysplasia	4	4.25
Grade I Atypical Transformation	48	51.06
Grade II Atypical Transformation	34	36.18
Suggestive of Carcinoma	8	8.51
Total	94	100

**Table 11:** Distribution of cases by histopathological examination.

Histopathological Examination	Number of Cases	Percentage (%)
Low-Grade CIN	46	48.94
High-Grade CIN	40	42.55
Invasive Cancer	8	8.51
Total	94	100

CIN = cervical intraepithelial neoplasia.

**Table 12:** Concordance between cervicovaginal cytology and histopathological diagnosis.

Cytological Examination	ASCUS	LSIL	HSIL	Cells Suggestive of Squamous Cancer	Number of Cases	Percentage (%)
Histopathological Examination						
Low-Grade CIN	14	26	6	0	46	48.94
High-Grade CIN	4	5	28	3	40	42.55
Squamous Cancer	0	1	2	5	8	8.51
Total	18	32	36	8	94	100

CIN = cervical intraepithelial neoplasia, ASCUS = atypical squamous cells of undetermined significance, LSIL = low-grade squamous intraepithelial lesion, HSIL = high-grade squamous intraepithelial lesion.

**Table 13:** Concordance between colposcopic findings and histopathological diagnosis.

Colposcopic Examination	Negative for Dysplasia	Grade I Atypical Transformation	Grade II Atypical Transformation	Suggestive of Invasive Cancer	Number of Cases	Percentage (%)
Low-Grade CIN	4	34	7	1	46	48.94
High-Grade CIN	0	14	22	4	40	42.55
Invasive Cancer	0	0	5	3	8	8.51
Total	4	48	34	8	94	100

CIN = cervical intraepithelial neoplasia.

## Data Availability

The data presented in this study are available upon request from the corresponding author.
